# Bis{2-[(5-hy­droxy­pent­yl)imino­meth­yl]phenolato-κ^2^ *N*,*O* ^1^}copper(II)

**DOI:** 10.1107/S1600536813016802

**Published:** 2013-06-26

**Authors:** Ritwik Modak, Santu Patra, Senjuti Mandal, Yeasin Sikdar, Sanchita Goswami

**Affiliations:** a Department of Chemistry, University of Calcutta, 92 A.P.C. Road, Kolkata 700 009, USA

## Abstract

In the title compound, [Cu(C_12_H_16_NO_2_)_2_], the Cu^II^ ion, located on a center of inversion, is coordinated by two singly deprotonated Schiff base ligands derived from condensation of salicyldehyde and 1-amino­pentan-5-ol. The imino N and phenol O atoms from both ligands offer a square-planar arrangement around the metal ion. The Cu—N and Cu—O bond lengths are 2.0146 (15) and 1.8870 (12) Å, respectively. Since the Cu—O and Cu—N bond lengths are different, it can be concluded that the resulting geometry of the complex is distorted. The aliphatic –OH group of the ligand is not coordinated and points away from the metal coordination zone and actively participates in hydrogen bonding connecting two other units and thus stabilizing the crystal lattice. This results in a two-dimensional extended array parallel to (201).

## Related literature

For the participation of the copper ion in the active sites of a large number of metalloproteins involved in important biological electron-transfer reactions, see: Reedijk & Bouwman (1999 ▶); Solomon *et al.* (2001 ▶); Hatcher & Karlin (2004 ▶); Kaim & Rall (1996 ▶). For references regarding the t_4_ value, see: Yang *et al.* (2007 ▶). For similar Cu—N and Cu—O bond lengths, see: Maeda *et al.* (2003 ▶); Akimova *et al.* (2001 ▶); Pawlicki *et al.* (2007 ▶); Verma *et al.* (2011 ▶); Khandar & Nejati (2000 ▶); Sundaravel *et al.* (2009 ▶).
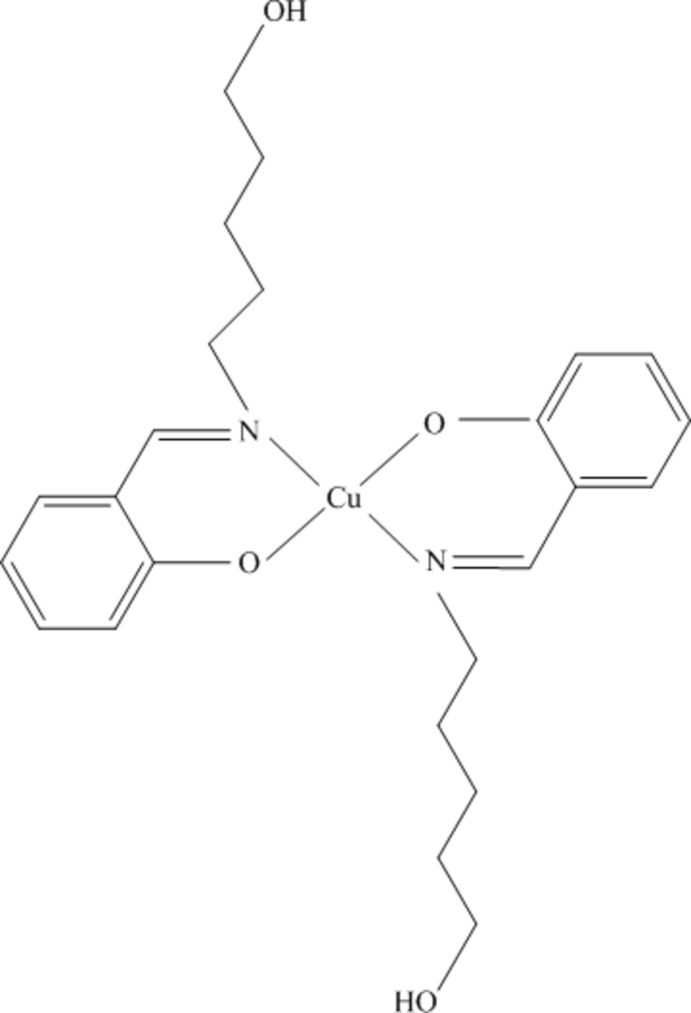


## Experimental

### 

#### Crystal data

[Cu(C_12_H_16_NO_2_)_2_]
*M* *_r_* = 476.07
Monoclinic, 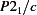

*a* = 11.8815 (8) Å
*b* = 5.2219 (3) Å
*c* = 18.9588 (12) Å
β = 102.876 (2)°
*V* = 1146.70 (12) Å^3^
*Z* = 2
Mo *K*α radiation
μ = 0.99 mm^−1^
*T* = 296 K
0.8 × 0.6 × 0.4 mm

#### Data collection

Bruker APEXII SMART CCD diffractometer
Absorption correction: multi-scan (*SADABS*; Sheldrick, 1996 ▶) *T*_min_ = 0.497, *T*_max_ = 0.674
13343 measured reflections
2549 independent reflections
2174 reflections with *I* > 2σ(*I*)
*R*_int_ = 0.027

#### Refinement

*R*[*F* ^2^ > 2σ(*F* ^2^)] = 0.030
*wR*(*F* ^2^) = 0.096
*S* = 0.95
2549 reflections
143 parameters
H-atom parameters constrained
Δρ_max_ = 0.27 e Å^−3^
Δρ_min_ = −0.30 e Å^−3^

### 

Data collection: *APEX2* (Bruker, 2004 ▶); cell refinement: *SAINT* (Bruker, 2004 ▶); data reduction: *SAINT*; program(s) used to solve structure: *SHELXS97* (Sheldrick, 2008 ▶); program(s) used to refine structure: *SHELXL97* (Sheldrick, 2008 ▶); molecular graphics: *ORTEP-3 for Windows* (Farrugia, 2012 ▶); software used to prepare material for publication: *Mercury* (Macrae *et al.*, 2008 ▶).

## Supplementary Material

Crystal structure: contains datablock(s) I, global. DOI: 10.1107/S1600536813016802/bv2221sup1.cif

Structure factors: contains datablock(s) I. DOI: 10.1107/S1600536813016802/bv2221Isup2.hkl

Additional supplementary materials:  crystallographic information; 3D view; checkCIF report

## Figures and Tables

**Table 1:** Hydrogen-bond geometry (Å, °)

*D*—H⋯*A*	*D*—H	H⋯*A*	*D*⋯*A*	*D*—H⋯*A*
O1—H1⋯O1^i^	0.82	2.07	2.864 (2)	163
C1—H1*B*⋯O2^ii^	0.97	2.34	2.771 (2)	106

Symmetry codes: (i) 
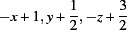
; (ii) 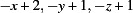.

**Fig. 1.**
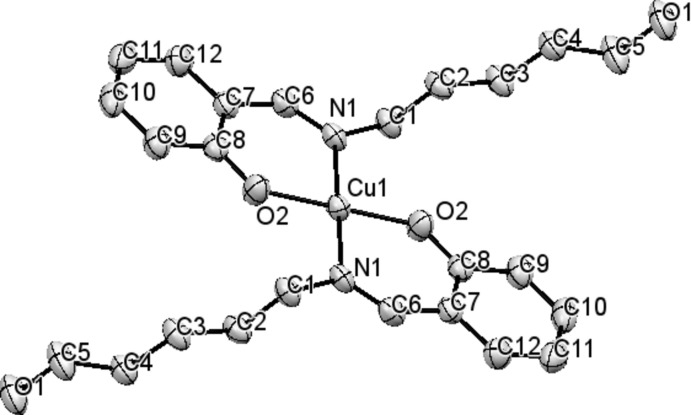
The structure of (I), showing the atom-labelling scheme. H atoms omitted for clarity. Displacement ellipsoids are drawn at the 50% probability level.

**Fig. 2.**
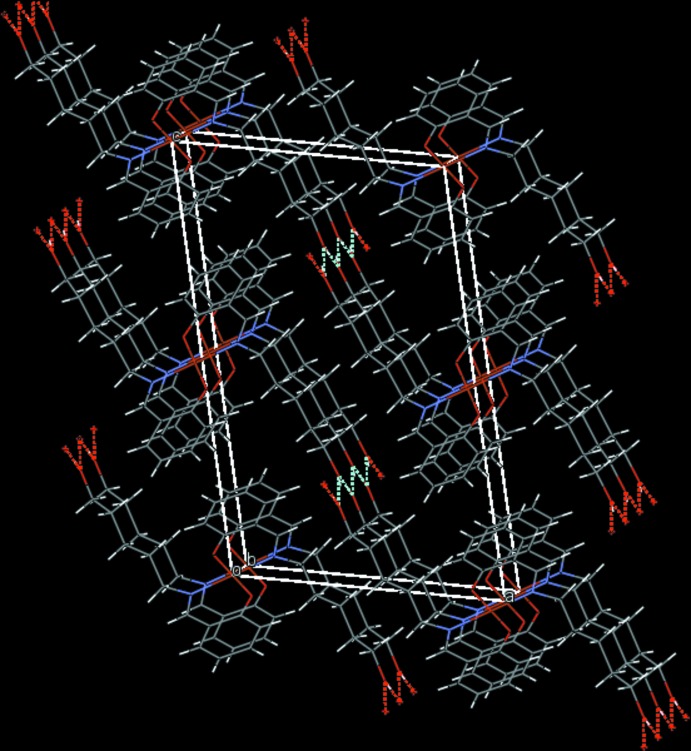
A view of the two-dimensional hydrogen-bonded framework viewed along the b axis. Hydrogen bonding interactions are shown by dashed lines.

## supplementary crystallographic information

### Comment

Coordination chemistry of copper complexes of chelating ligands is a subject of continuing importance in connection with their structural, spectral, and redox properties in general and from the standpoint of their relevance to copper-containing metalloproteins in particular (Solomon *et al.*, 2001; Hatcher & Karlin, 2004; Kaim & Rall, 1996). Copper ions are found in the active sites of a large number of metalloproteins involved in important biological electron-transfer reactions, as well as in redox processes of molecular oxygen (Reedijk & Bouwman, 1999).

Crystallographic analysis reveals that the asymmetric unit of the title mononuclear complex consists of one Cu^II^ ion, which is located on a center of inversion, and two singly deprotonated ligands, HL^-^, with the phenolic O atom being deprotonated. The phenolic O atoms (O2 and O2_a; symmetry code: (*a*) 2-*x*, 1-*y*, 1-*z*) and the imine N atoms (N1 and N1_a; symmetry code: (*a*) 2-*x*, 1-*y*, 1-*z*) from both the ligands coordinate to the same Cu^II^ center in the *trans* disposition to each other. The aliphatic –OH group remains as a pendant arm and is pointing away from the metal coordination zone. This uncoordinated oxygen atom, O1, is 8.083 Å away from the Cu^II^ ion. The complex has a τ_4_ value of 0 (α = O2 - Cu1 - O2_a = 180.00 and β = N1 - Cu1 - N1_a = 180.00) as a consequence of the Cu lying on a center of inversion thus supporting an assignment of distorted square planar geometry around the central metal ion (Yang *et al.* 2007). The complex exhibits a Cu1 – N1 bond length of 2.0146 (16) Å. In a perfectly square planar CuN_4_ moiety, the average Cu^II^ – N distance lies in the range of 1.980 (9) and 2.018 (9) Å (Maeda *et al.*,2003, Akimova *et al.*, 2001). The Cu – N bond length value is comparable to the previously reported nearly planar Cu^II^ porphyrins (2.020 Å, 2.065 Å, 1.977 Å) (Pawlicki *et al.* 2007). It agrees well with the CuN_2_O_2_ monomer (τ_4_ = 1/5) having average Cu^II^ – N bond length range of 2.071 Å (Verma *et al.*, 2011). The Cu1 – O2 bond distance in the complex is 1.8871 (11) Å. It is well established in the literature that in a nearly square planar geometry, the Cu^II^ – phenolic oxygen bond length lies in the range of 1.84 Å to 1.93 Å (Khandar & Nejati, 2000; Sundaravel *et al.*, 2009). Since the Cu - O and Cu - N bond lengths are different, therefore, it can be concluded that the resultant geometry is a distorted square planar one. The pendant –OH group actively participates in H-bonding and connects two other units stabilizing the crystal lattice. As a result we have a two-dimensional extended array parallel to 201 plane with O1 - H1 - - - O1 length 2.864 (2) Å.

### Experimental

The solution of 5-amino-1-pentanol (3 mmol, 650.8 mg) in methanol (20 mL) was added to the solution of salicylaldehyde (3 mmol, 366.36 mg) in methanol (20 ml) under vigorous stirring condition. The resulting reaction mixture was subsequently refluxed with stirring for 4 h. Completion of the reaction checked by thin layer chromatography (TLC). After reaction was complete, the solution was dried over Na_2_SO_4_, followed by filtration and the solvent was removed under reduced pressure to get the ligand. Now a solution of Cu(OAc)_2_.H_2_O (1.5 mmol, 299.47 mg) in methanol (20 ml) was added to the solution of the prepared crude ligand (3 mmol, 621.84 mg) in methanol(20 ml) with constant stirring. The resulting mixture was stirred for 3 h at room temperature and then filtered. The resulting dark brown solution on slow evaporation gave a brown amorphous solid which was washed with diethyl ether properly and dried in vacuum desiccator containing anhydrous CaCl_2_. X-ray quality single crystals were grown from acetonitrile by the slow evaporation method.

### Refinement

The H atoms were placed in calculated positions and refined as riding atoms, with C—H = 0.93 Å, aliphatic C – H = 0.97 Å and O – H = 0.82 Å.

### Crystal data

**Table:**

[Cu(C_12_H_16_NO_2_)_2_]	*F*(000) = 502.0
*M**_r_* = 476.07	*D*_x_ = 1.385 Mg m^−^^3^
Monoclinic, *P*2_1_/*c*	Mo *K*α radiation, λ = 0.71073 Å
Hall symbol: -P 2ybc	Cell parameters from 13343 reflections
*a* = 11.8815 (8) Å	θ = 1.8–27.5°
*b* = 5.2219 (3) Å	µ = 0.99 mm^−^^1^
*c* = 18.9588 (12) Å	*T* = 296 K
β = 102.876 (2)°	Block, dark green
*V* = 1146.70 (12) Å^3^	0.8 × 0.6 × 0.4 mm
*Z* = 2	

### Data collection

**Table:**

Bruker APEXII SMART CCD diffractometer	2549 independent reflections
Radiation source: fine-focus sealed tube	2174 reflections with *I* > 2σ(*I*)
Graphite monochromator	*R*_int_ = 0.027
φ and ω scans	θ_max_ = 27.5°, θ_min_ = 1.8°
Absorption correction: multi-scan (*SADABS*; Sheldrick, 1996)	*h* = −14→14
*T*_min_ = 0.497, *T*_max_ = 0.674	*k* = −6→6
13343 measured reflections	*l* = −24→24

### Refinement

**Table:**

Refinement on *F*^2^	Primary atom site location: structure-invariant direct methods
Least-squares matrix: full	Secondary atom site location: difference Fourier map
*R*[*F*^2^ > 2σ(*F*^2^)] = 0.030	Hydrogen site location: inferred from neighbouring sites
*wR*(*F*^2^) = 0.096	H-atom parameters constrained
*S* = 0.95	*w* = 1/[σ^2^(*F*_o_^2^) + (0.0695*P*)^2^ + 0.3017*P*] where *P* = (*F*_o_^2^ + 2*F*_c_^2^)/3
2549 reflections	(Δ/σ)_max_ = 0.015
143 parameters	Δρ_max_ = 0.27 e Å^−^^3^
0 restraints	Δρ_min_ = −0.30 e Å^−^^3^

### Special details

**Table:**

Geometry. All e.s.d.'s (except the e.s.d. in the dihedral angle between two l.s. planes) are estimated using the full covariance matrix. The cell e.s.d.'s are taken into account individually in the estimation of e.s.d.'s in distances, angles and torsion angles; correlations between e.s.d.'s in cell parameters are only used when they are defined by crystal symmetry. An approximate (isotropic) treatment of cell e.s.d.'s is used for estimating e.s.d.'s involving l.s. planes.
Refinement. Refinement of *F*^2^ against ALL reflections. The weighted *R*-factor *wR* and goodness of fit *S* are based on *F*^2^, conventional *R*-factors *R* are based on *F*, with *F* set to zero for negative *F*^2^. The threshold expression of *F*^2^ > σ(*F*^2^) is used only for calculating *R*-factors(gt) *etc*. and is not relevant to the choice of reflections for refinement. *R*-factors based on *F*^2^ are statistically about twice as large as those based on *F*, and *R*- factors based on ALL data will be even larger.

### Fractional atomic coordinates and isotropic or equivalent isotropic displacement parameters (Å^2^)

**Table:**

	*x*	*y*	*z*	*U*_iso_*/*U*_eq_	
Cu1	1.0000	0.5000	0.5000	0.03217 (12)	
O1	0.50800 (15)	0.8210 (3)	0.72046 (8)	0.0640 (4)	
H1	0.4971	0.9476	0.7436	0.096*	
O2	1.04519 (10)	0.2230 (2)	0.44865 (7)	0.0440 (3)	
N1	0.83417 (13)	0.4686 (3)	0.44597 (8)	0.0338 (3)	
C1	0.74512 (14)	0.6417 (3)	0.46237 (9)	0.0372 (4)	
H1A	0.6794	0.6453	0.4215	0.045*	
H1B	0.7761	0.8140	0.4697	0.045*	
C2	0.70561 (16)	0.5564 (3)	0.52942 (10)	0.0389 (4)	
H2A	0.6628	0.3975	0.5191	0.047*	
H2B	0.7727	0.5236	0.5680	0.047*	
C3	0.63016 (16)	0.7546 (4)	0.55460 (10)	0.0420 (4)	
H3A	0.5621	0.7829	0.5163	0.050*	
H3B	0.6722	0.9150	0.5629	0.050*	
C4	0.59209 (16)	0.6804 (4)	0.62336 (10)	0.0422 (4)	
H4A	0.5346	0.5457	0.6122	0.051*	
H4B	0.6579	0.6127	0.6581	0.051*	
C5	0.5428 (2)	0.8995 (5)	0.65661 (12)	0.0571 (5)	
H5A	0.6001	1.0342	0.6685	0.068*	
H5B	0.4768	0.9679	0.6222	0.068*	
C6	0.79987 (14)	0.3133 (3)	0.39297 (9)	0.0375 (4)	
H6	0.7230	0.3277	0.3687	0.045*	
C7	0.86641 (14)	0.1202 (3)	0.36685 (8)	0.0362 (4)	
C8	0.98489 (15)	0.0831 (3)	0.39729 (9)	0.0357 (3)	
C9	1.04061 (17)	−0.1203 (3)	0.36855 (10)	0.0437 (4)	
H9	1.1187	−0.1500	0.3873	0.052*	
C10	0.98222 (18)	−0.2730 (4)	0.31404 (10)	0.0485 (5)	
H10	1.0212	−0.4049	0.2967	0.058*	
C11	0.86595 (19)	−0.2348 (4)	0.28419 (10)	0.0502 (5)	
H11	0.8270	−0.3392	0.2469	0.060*	
C12	0.80936 (19)	−0.0411 (4)	0.31035 (11)	0.0452 (4)	
H12	0.7313	−0.0149	0.2904	0.054*	

### Atomic displacement parameters (Å^2^)

**Table:**

	*U*^11^	*U*^22^	*U*^33^	*U*^12^	*U*^13^	*U*^23^
Cu1	0.02804 (18)	0.03330 (18)	0.03728 (18)	0.00376 (10)	0.01180 (12)	−0.00224 (10)
O1	0.0847 (11)	0.0657 (9)	0.0553 (8)	0.0084 (8)	0.0448 (8)	0.0046 (7)
O2	0.0337 (6)	0.0444 (7)	0.0535 (7)	0.0065 (5)	0.0088 (5)	−0.0123 (6)
N1	0.0299 (7)	0.0393 (8)	0.0358 (7)	0.0058 (5)	0.0146 (6)	0.0026 (5)
C1	0.0309 (8)	0.0425 (9)	0.0405 (8)	0.0106 (7)	0.0131 (6)	0.0037 (7)
C2	0.0356 (9)	0.0362 (8)	0.0498 (10)	0.0068 (7)	0.0203 (7)	0.0052 (7)
C3	0.0427 (9)	0.0422 (9)	0.0471 (9)	0.0112 (7)	0.0226 (8)	0.0079 (7)
C4	0.0442 (10)	0.0426 (9)	0.0444 (9)	0.0038 (7)	0.0198 (7)	0.0042 (7)
C5	0.0746 (15)	0.0529 (12)	0.0562 (12)	0.0147 (11)	0.0415 (11)	0.0116 (10)
C6	0.0312 (8)	0.0467 (9)	0.0359 (8)	0.0016 (7)	0.0103 (6)	0.0029 (7)
C7	0.0402 (9)	0.0369 (9)	0.0350 (8)	−0.0010 (7)	0.0158 (7)	0.0006 (7)
C8	0.0400 (9)	0.0318 (8)	0.0386 (8)	0.0028 (7)	0.0161 (7)	0.0015 (7)
C9	0.0481 (10)	0.0371 (9)	0.0486 (10)	0.0099 (8)	0.0167 (8)	−0.0001 (8)
C10	0.0693 (13)	0.0356 (9)	0.0464 (10)	0.0071 (9)	0.0252 (9)	−0.0032 (8)
C11	0.0651 (13)	0.0474 (10)	0.0399 (9)	−0.0069 (9)	0.0156 (8)	−0.0066 (8)
C12	0.0459 (11)	0.0537 (11)	0.0369 (9)	−0.0038 (8)	0.0110 (8)	−0.0025 (7)

### Geometric parameters (Å, º)

**Table:**

Cu1—O2^i^	1.8870 (12)	C4—C5	1.488 (3)
Cu1—O2	1.8870 (12)	C4—H4A	0.9700
Cu1—N1^i^	2.0146 (15)	C4—H4B	0.9700
Cu1—N1	2.0146 (15)	C5—H5A	0.9700
O1—C5	1.424 (2)	C5—H5B	0.9700
O1—H1	0.8200	C6—C7	1.436 (2)
O2—C8	1.298 (2)	C6—H6	0.9300
N1—C6	1.285 (2)	C7—C8	1.411 (2)
N1—C1	1.476 (2)	C7—C12	1.411 (3)
C1—C2	1.517 (2)	C8—C9	1.423 (2)
C1—H1A	0.9700	C9—C10	1.366 (3)
C1—H1B	0.9700	C9—H9	0.9300
C2—C3	1.514 (2)	C10—C11	1.386 (3)
C2—H2A	0.9700	C10—H10	0.9300
C2—H2B	0.9700	C11—C12	1.368 (3)
C3—C4	1.522 (2)	C11—H11	0.9300
C3—H3A	0.9700	C12—H12	0.9300
C3—H3B	0.9700		
			
O2^i^—Cu1—O2	179.999 (1)	C3—C4—H4A	109.0
O2^i^—Cu1—N1^i^	91.94 (5)	C5—C4—H4B	109.0
O2—Cu1—N1^i^	88.06 (5)	C3—C4—H4B	109.0
O2^i^—Cu1—N1	88.06 (5)	H4A—C4—H4B	107.8
O2—Cu1—N1	91.94 (5)	O1—C5—C4	110.77 (17)
N1^i^—Cu1—N1	179.998 (1)	O1—C5—H5A	109.5
C5—O1—H1	109.5	C4—C5—H5A	109.5
C8—O2—Cu1	130.21 (11)	O1—C5—H5B	109.5
C6—N1—C1	115.71 (15)	C4—C5—H5B	109.5
C6—N1—Cu1	123.56 (12)	H5A—C5—H5B	108.1
C1—N1—Cu1	120.58 (11)	N1—C6—C7	127.59 (15)
N1—C1—C2	111.53 (13)	N1—C6—H6	116.2
N1—C1—H1A	109.3	C7—C6—H6	116.2
C2—C1—H1A	109.3	C8—C7—C12	119.69 (16)
N1—C1—H1B	109.3	C8—C7—C6	122.08 (15)
C2—C1—H1B	109.3	C12—C7—C6	118.20 (16)
H1A—C1—H1B	108.0	O2—C8—C7	124.29 (15)
C3—C2—C1	112.27 (14)	O2—C8—C9	118.78 (16)
C3—C2—H2A	109.2	C7—C8—C9	116.92 (16)
C1—C2—H2A	109.2	C10—C9—C8	121.64 (18)
C3—C2—H2B	109.2	C10—C9—H9	119.2
C1—C2—H2B	109.2	C8—C9—H9	119.2
H2A—C2—H2B	107.9	C9—C10—C11	121.12 (17)
C2—C3—C4	113.92 (15)	C9—C10—H10	119.4
C2—C3—H3A	108.8	C11—C10—H10	119.4
C4—C3—H3A	108.8	C12—C11—C10	118.98 (18)
C2—C3—H3B	108.8	C12—C11—H11	120.5
C4—C3—H3B	108.8	C10—C11—H11	120.5
H3A—C3—H3B	107.7	C11—C12—C7	121.66 (19)
C5—C4—C3	112.76 (15)	C11—C12—H12	119.2
C5—C4—H4A	109.0	C7—C12—H12	119.2

Symmetry code: (i) −*x*+2, −*y*+1, −*z*+1.

### Hydrogen-bond geometry (Å, º)

**Table:**

*D*—H···*A*	*D*—H	H···*A*	*D*···*A*	*D*—H···*A*
O1—H1···O1^ii^	0.82	2.07	2.864 (2)	163
C1—H1*B*···O2^i^	0.97	2.34	2.771 (2)	106

Symmetry codes: (i) −*x*+2, −*y*+1, −*z*+1; (ii) −*x*+1, *y*+1/2, −*z*+3/2.
